# Bioactive Ingredients for Safe and Health-Promoting Functional Foods

**DOI:** 10.3390/foods12224134

**Published:** 2023-11-15

**Authors:** Fernanda Maria Vanin, Rosemary Aparecida de Carvalho, Vitor Augusto Dos Santos Garcia, Cristiana Maria Pedroso Yoshida

**Affiliations:** 1 Laboratory of Bread and Dough Process (LAPROPAMA), Food Engineering Department, Faculty of Animal Science and Food Engineering (USP/FZEA), University of São Paulo, Av. Duque de Caxias Norte 225, Pirassununga 13635-900, SP, Brazil; 2 Faculty of Animal Science and Food Engineering, University of São Paulo, Av. Duque de Caxias Norte, 225, Pirassununga 13635-900, SP, Brazil; rosecarvalho@usp.br; 3 Faculty of Agricultural Sciences, UNESP—São Paulo State University, Av. Universitária, 3780, Botucatu 18610-034, SP, Brazil; vitor.as.garcia@unesp.br; 4 Institute of Environmental, Chemical and Pharmaceutical Sciences, Universidade Federal de São Paulo (UNIFESP), Rua São Nicolau, 210, Diadema 09913-030, SP, Brazil; cristiana.yoshida@unifesp.br

## 1. Introduction

A full ingredient or a portion of food utilized as food for specific therapeutic purposes is referred to as a functional food [1]. In order to create safe and healthy functional foods, bioactive ingredients are essential. These bioactive compounds are naturally founded in different foods; however, in order to increase their consumption, they could be included in food formulations.

As an example of bioactive compounds, antioxidants, probiotics, prebiotics, phenolic compounds, fatty acids, phytosterols, fiber, polyunsaturated fats, and phytochemicals, among others, may be cited [1,2].

However, for the safe use of bioactive ingredients in the formulation of functional foods, studies related to ensuring the aspects of safety, stability, and efficacy are also essential. Clinical studies and regulatory approvals may be necessary to support health claims associated with these ingredients, and they should be used in appropriate concentrations to provide the desired health benefits without adverse effects. Additionally, consumers should be informed about the presence and benefits of bioactive ingredients in functional foods to make informed dietary choices.

## 2. Food Security, Food Waste, Food Safety, and Food Engineering

According to the « Food and Agriculture Organization of the United Nations » (FAO), global hunger and the prevalence of severe food malnutrition have grown worldwide, especially in recent years [3]. Additionally, in 2050, the world’s population will have increased by 34%, and to supply this, food production needs to increase by 70%. On the other hand, approximately on third of all food produced for human consumption is lost or wasted annually. Losses in the production, post-harvest, and processing stages may be responsible for more than 40% of total losses in some regions of the world such as Africa and Latin America [4].

The world population has been increasing alongside global hunger and malnutrition, particularly over the last five years. Furthermore, the novel coronavirus (COVID-19) has impacted several aspects of food and nutrition security, especially in regions of extreme hunger and food insecurity.

Moreover, the World Health Organization estimates that 600 million people suffer from foodborne diseases after eating contaminated food, and 420,000 die every year for this reason [5]. Foodborne illnesses are typically brought on by microbiological contamination through bacteria, viruses, parasites, or chemical contamination, which could be due to natural compounds present in foods or persistent organic pollutants, pesticide residues, heavy metals, etc., which enter the human system via food intake. 

All these factors underline the need for new strategies to reduce food insecurity and food waste, and enhance food safety in the food chain using more sustainable practices; these practices are applied with the aim of implementing a circular economy in the food industry. 

In the principle of sustainable food production, the use of innovative and different technologies is a matter of great relevance. The use of ingredients from by-products and/or residues in the food industry is still rare. Several studies have shown that these residues, for the most part, are rich in nutrients; and could, they could thus add nutritional and functional value to different products.

## 3. Considerations for the Development of Ingredients for Food Safety and Health Promotion

If the use of waste and residues from fruits, legumes, and vegetables allows for the production of food products with improved nutritional composition, such as higher levels of resistant starch, dietary fiber, bioactive compounds, minerals, and others, the effect of these materials on digestion has still been scarcely evaluated. One example of such products is peach palm flour, an Amazonian fruit that has been widely studied and incorporated into different bakery products, such as bread, cakes and cookies. However, a recent study demonstrated that peach palm flour impacted protein digestibility in a food model, and also produced cytotoxicity in normal cells [6].

Therefore, the aim of this Special Issue is to collate studies that could represent solutions to these multifaceted and dynamic problems, holistically contemplating their multiple facets. Studies related to food engineering strategies for producing bioactive ingredients, related to innovative alternative methods of reducing food insecurity, and related to increasing health and safety promotion are particularly welcome.

The development and understanding of the effect of bioactive ingredients on the different aspects of food production is subject that must be evaluated to enhance the production of safe and health-promoting foods.

## References

[1] da Silva B.V., Barreira J.C., Oliveira M.B.P. (2016). Natural phytochemicals and probiotics as bioactive ingredients for functional foods: Extraction, biochemistry and protected-delivery technologies. Trends Food Sci. Technol..

[2] Fernandes S.S., Coelho M.S., de las Mercedes Salas-Mellado M. (2019). Bioactive compounds as ingredients of functional foods: Polyphenols, carotenoids, peptides from animal and plant sources new. Bioactive Compounds.

[3] FAO, IFAD, UNICEF, WFP, WHO (2023). The State of Food Security and Nutrition in the World 2023. Urbanization, Agrifood Systems Transformation and Healthy Diets across the Rural–Urban Continuum.

[4] (2011). LOSSES, FAO Global Food. Waste, Food. Extent, Causes and Prevention.

[5] Food Safety. https://www.who.int/news-room/fact-sheets/detail/food-safety#:~:text=An%20estimated%20600%20million%20%E2%80%93%20almost,healthy%20life%20years%20(DALYs).

[6] Santos Y.J.S., Facchinatto W.M., Rochetti A.L., Carvalho R.A., Le Feunteun S., Fukumasu H., Vanin F.M. (2023). Systemic characterization of pupunha (*Bactris gasipaes*) flour with views of polyphenol content on cytotoxicity and protein in vitro digestion. Food Chem..

